# Impact of the COVID-19 Pandemic on Antibiotic Prescribing for Common Infections in The Netherlands: A Primary Care-Based Observational Cohort Study

**DOI:** 10.3390/antibiotics10020196

**Published:** 2021-02-18

**Authors:** Alma C. van de Pol, Josi A. Boeijen, Roderick P. Venekamp, Tamara Platteel, Roger A. M. J. Damoiseaux, Marlous F. Kortekaas, Alike W. van der Velden

**Affiliations:** Julius Center for Health Sciences and Primary Care, University Medical Center Utrecht, Utrecht University, Heidelberglaan 100, 3584 CX Utrecht, The Netherlands; A.C.vandePol-11@umcutrecht.nl (A.C.v.d.P.); R.P.Venekamp@umcutrecht.nl (R.P.V.); T.N.Platteel-3@umcutrecht.nl (T.P.); R.A.M.J.Damoiseaux@umcutrecht.nl (R.A.M.J.D.); mkortekaas@lrjg.nl (M.F.K.); a.w.vandervelden@umcutrecht.nl (A.W.v.d.V.)

**Keywords:** COVID-19, pandemic, antibiotic, infectious disease, respiratory tract infection, urinary tract infection, pneumonia, routine care data, complications

## Abstract

In 2020, the COVID-19 pandemic brought dramatic changes in the delivery of primary health care across the world, presumably changing the number of consultations for infectious diseases and antibiotic use. We aimed to assess the impact of the pandemic on infections and antibiotic prescribing in Dutch primary care. All patients included in the routine health care database of the Julius General Practitioners’ Network were followed from March through May 2019 (*n* = 389,708) and March through May 2020 (*n* = 405,688). We extracted data on consultations for respiratory/ear, urinary tract, gastrointestinal and skin infections using the International Classification of Primary Care (ICPC) codes. These consultations were combined in disease episodes and linked to antibiotic prescriptions. The numbers of infectious disease episodes (total and those treated with antibiotics), complications, and antibiotic prescription rates (i.e., proportion of episodes treated with antibiotics) were calculated and compared between the study periods in 2019 and 2020. Fewer episodes were observed during the pandemic months than in the same months in 2019 for both the four infectious disease entities and complications such as pneumonia, mastoiditis and pyelonephritis. The largest decline was seen for gastrointestinal infections (relative risk (RR), 0.54; confidence interval (CI), 0.51 to 0.58) and skin infections (RR, 0.71; CI, 0.67 to 0.75). The number of episodes treated with antibiotics declined as well, with the largest decrease seen for respiratory/ear infections (RR, 0.54; CI, 0.52 to 0.58). The antibiotic prescription rate for respiratory/ear infections declined from 21% to 13% (difference −8.0% (CI, −8.8 to −7.2)), yet the prescription rates for other infectious disease entities remained similar or increased slightly. The decreases in primary care infectious disease episodes and antibiotic use were most pronounced in weeks 15–19, mid-COVID-19 wave, after an initial peak in respiratory/ear infection presentation in week 11, the first week of lock-down. In conclusion, our findings indicate that the COVID-19 pandemic has had profound effects on the presentation of infectious disease episodes and antibiotic use in primary care in the Netherlands. Consequently, the number of infectious disease episodes treated with antibiotics decreased. We found no evidence of an increase in complications.

## 1. Introduction

The COVID-19 pandemic has brought dramatic changes in the delivery of primary health care, especially for patients with respiratory tract infections [1,2]. The pandemic prompted an unprecedented restructuring of primary care practice, resulting in a rapid switch from face-to-face to remote consultations. It has been suggested that virtual assessments limit diagnostic capabilities and could thereby result in overprescribing of antibiotics [3,4]. The latter is of particular concern, since this has been recognized as the main driver of antimicrobial resistance [5,6]. In the past decade, many countries implemented antibiotic surveillance and stewardship programs to promote the judicious use of antibiotics to combat resistance, also in the primary care setting where the vast majority of antibiotics for human use are issued [7].

Specifically with respect to antibiotic prescribing, numerous interesting phenomena may have intertwined during the 2020 COVID-19 pandemic. Antibiotic use depends on the number of patients in the community experiencing infections, patients’ consultation behavior (do they consult their general practitioner (GP), and if so, face-to-face or remotely), and GPs’ prescribing behavior. Several issues during the pandemic could have led to an increase in antibiotic prescriptions. First, patients may have experienced more respiratory tract symptoms during the pandemic due to circulating SARS-CoV-2 beside other respiratory pathogens. Second, patients may have consulted their GP more often for COVID-19-related anxiety and/or reassurance. Finally, GPs may have lowered their antibiotic prescribing threshold as a result of diagnostic uncertainty during remote consultation and to prevent adverse outcomes whilst there is limited hospital capacity. On the contrary, the incidence of infections may have lowered due to social distancing, hand washing, school closure and quarantine measures (starting in week 11 in the Netherlands). Additionally, patients may have been less inclined to consult their GP (as a result of advice to stay away from general practices and self-manage uncomplicated infectious conditions), and GPs may have more frequently perceived respiratory tract infections as being of viral origin, thus without a need for antibiotics, due to the introduction of SARS-CoV-2.

Since so many factors may have altered antibiotic use, we set out to evaluate the actual impact of the COVID-19 pandemic on the number of GP registered episodes and antibiotic prescribing for common infections in primary care in the Netherlands by comparing routine care data from the first wave of the COVID-19 pandemic in 2020 to those from the same period one year earlier.

## 2. Results

### 2.1. Study Population

From March through May 2019 and 2020, 389,708 and 405,688 patients (49% male) were registered in the Julius General Practitioners’ Network (JGPN) practices, respectively. In 2019, 40,219 consultations were extracted, which related to 27,263 infectious disease episodes. In 2020, 37,604 consultations and 23,442 related episodes were found. The overall antibiotic prescription rate was 27% in 2019 and 23% in 2020.

### 2.2. Number of Common Infectious Disease Episodes, in Total and with Antibiotic Prescription

Table 1 shows that for all four infectious disease entities fewer episodes were observed in primary care during the pandemic than in the year before. The decline was largest for gastrointestinal infections (RR, 0.54; CI, 0.51 to 0.58) and skin infections (RR, 0.71; CI, 0.67 to 0.75), but was also apparent for respiratory/ear infections (RR, 0.90; CI, 0.88 to 0.92). Concomitantly, the absolute number of episodes treated with antibiotics decreased for the four infectious disease entities, with the largest decrease seen for respiratory/ear infections (RR, 0.54; CI, 0.52 to 0.58).

The antibiotic prescription rate for respiratory/ear infections was most affected by the pandemic, decreasing from 21% to 13% (−8% (CI, −8.8% to −7.2%)).

### 2.3. Number of Infectious Disease Episodes and Episodes with Antibiotic Prescription during the Pandemic Weeks

**Figure 1:**
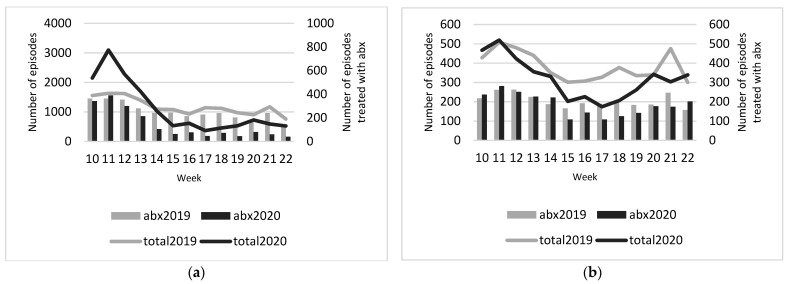

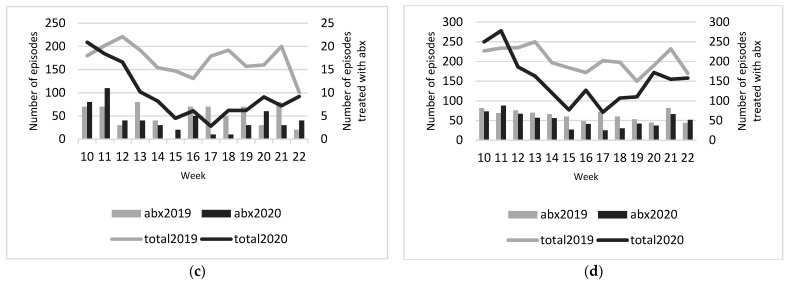
Total number of infectious disease episodes and episodes with antibiotic prescription in 2019 and 2020, per week, (**a**) respiratory/ear infections, (**b**) urinary tract infection, (**c**) gastrointestinal infections, (**d**) skin infections. Abbreviations: Abx: antibiotics.

Figure 1 shows that the number of infectious disease episodes highly fluctuated over time. Particularly the number of respiratory/ear infection episodes increased substantially early in the pandemic, with a peak in week 11 (first week of lock-down), and from then on decreased to a level below that of 2019 from week 15 onwards. The number of episodes related to the other types of infections showed a more rapid decrease and were below the levels of 2019 from week 11 onwards.

The decreases in antibiotic prescribing followed similar patterns. The largest differences in antibiotic prescribing between 2019 and 2020 were observed for respiratory/ear infections from week 15 onwards and for the other three infection types for weeks 17 and 18.

### 2.4. Respiratory Tract and Ear Infections: Differences between Age Groups

A notable decrease in respiratory/ear infection episodes was observed in the youngest and oldest age categories (RR, 0.61; CI, 0.58 to 0.64 and RR, 0.82; CI, 0.78 to 0.86, respectively), whereas in 41–65-year-olds a slight increase was seen (RR, 1.14; CI, 1.10 to 1.19; Table 2). However, antibiotic prescribing for respiratory/ear infections decreased in all age groups, with the largest decrease in those aged 0–40 years. As a consequence, the antibiotic prescription rate decreased in all age categories.

Figures illustrating the numbers of respiratory/ear infection episodes over time (weeks) for the different age categories are shown in Supplementary Figures S1–S4. The initial peak in respiratory/ear infection episodes starting week 11 (first week of lockdown) in the 13–65-year-olds was higher and longer than in the 0–12 and >65-year-olds.

### 2.5. Analysis of Specific Diagnoses, Including Complications

The number of episodes with cough remained similar, whereas the number of episodes with influenza-like-illness increased. The number of bronchitis/bronchiolitis, acute otitis media (AOM) and cystitis episodes decreased (Table 3).

Acute upper respiratory tract infections (the code that could have been used for patients with suspected COVID-19) and other respiratory tract infections (the code to be used for confirmed COVID-19 in 2020) both increased.

The number of episodes of potential complications, i.e., pneumonia, mastoiditis and pyelonephritis, decreased or remained similar (Table 4).

The prescription rates for respiratory tract infection diagnoses, including codes used for (possible) COVID-19 infection, decreased. For AOM and cystitis prescription rates remained similar.

### 2.6. Remote-Only GP Consultations for Respiratory Tract and Ear Infections

From the 16,672 respiratory/ear infection episodes in 2019, 1907 (11.4%) consisted of one or more telephone consultations without a face-to-face consultation. This was 45.1% (7025 of 15,580) in 2020, which is a 4-fold increase. In 2019, in 5.5% (104/1907) of phone consultations an antibiotic was prescribed, which was only slightly higher, 6.1%, in the pandemic months (430/7025).

## 3. Discussion

Our observational cohort study reveals that the COVID-19 pandemic led to a reduction in the number of respiratory/ear, urinary tract, gastrointestinal and skin infections for which a GP was consulted, and in the number of episodes treated with antibiotics. The antibiotic prescription rate (i.e., proportion treated with antibiotics) for respiratory/ear infections declined from 21 to 13% (−8% (CI, −8.8% to −7.2%)) and remained similar or increased slightly for the other infectious disease entities. We found no evidence of an increase in complications such as pneumonia, mastoiditis, or pyelonephritis.

Reductions in presentation for common infections due to the COVID-19 pandemic were also found in other countries. The decrease in number of common infectious disease episodes for which a GP was consulted (from 27,263 to 23,442; −14%) in our study is slightly less pronounced than the reduction in overall number of primary care consultations (−21%) in the United Kingdom [8]. In the United States, a 40% relative reduction in childhoodAOM) incidence in primary and secondary care was observed [9]. We found a slightly larger reduction in primary care childhood AOM episodes in the Netherlands (RR, 0.37; CI, 0.34 to 0.41).

When looking at AOM specifically, we observed a decrease in patients consulting their GP for AOM during the pandemic, without an increase in prescription rate. This suggests that the case-mix of patients (i.e., the severity of AOM symptoms with which patients presented) remained largely unchanged. It is plausible that due to social distancing the incidence of respiratory infections, including AOM, in the community has dropped, explaining the lower number of AOM episodes in primary care. The incidence of urinary tract infections is not influenced by social distancing, therefore, the decrease in patients presenting at the GP for urinary tract infections is probably a result of discouraging patients to visit their GP for uncomplicated infections.

Changes in consultation behavior seem to depend on age. A decrease in respiratory/ear infection episodes was apparent in the youngest and oldest age categories, whereas in the 41–65-year-olds a slight increase was seen. Possibly, elderly and parents of children were more reluctant to contact their GPs per the advice to stay away from general practices and self-manage uncomplicated conditions. Patients aged 41–65 years may have contacted their GP for concerns about possible COVID-19 symptoms more frequently.

Contrary to the results of our study, where we found a decrease in the number of infection episodes for which antibiotics were prescribed, in UK primary care an unexpectedly high rate of antibiotic prescribing was observed during the COVID-19 pandemic [8]. In the UK this rise in antibiotic prescribing has been attributed to the increase in remote consultations [8]. A recent systematic review expressed concern about studies in the past (all performed pre-pandemic) indicating higher prescription rates in remote than in face-to-face consultations [10]. Although we did find an increase in remote consultations during the pandemic similar to the UK (+268% in the Netherlands and +270% in the UK [8]), we did not find an increase in antibiotic use.

Antibiotic use per inhabitant markedly varies between European countries; the Netherlands ranks among the lowest [11]. As our data revealed that the pandemic resulted in a further reduction in a low-use country, it is relevant to see how the pandemic influenced antibiotic prescribing in middle- and high-use countries. In a yearly point prevalence audit survey evaluating the management of respiratory tract infections in the PREPARE primary care research network, we found that antibiotic prescription rates dropped in 13 out of the 16 participating countries, but rose in the UK, Greece and Poland in the beginning of the pandemic as compared to the months just before the pandemic (personal communication, Alike van der Velden). In addition to these prescription rates, numbers of infection episodes for which the GP was consulted during the full first pandemic wave are needed to get the full picture of how GPs responded to the pandemic circumstances in individual countries with their specific context. If differential responses become apparent, insight from social science research could help elucidate reasons for these differences. Fields of exploration might be remote versus face-to-face consultations, countries’ guidelines for respiratory tract infections or COVID-19 management during the pandemic, patients’ health care seeking behavior and anxiety, instructions or safety directives of the management or professional organizations, reduced use of point-of-care diagnostics, or any other.

Closely related to differences in antibiotic use, levels of antimicrobial resistance vary concordantly between European countries [12]. Worldwide, decreasing antibiotic prescribing for self-limiting and often viral respiratory tract infections, and decreasing non-first choice prescribing is regarded key in fighting resistance development. Many multi-factorial interventions and programs are initiated to this aim in the primary care setting [13]. It is interesting to investigate how the observed reduction in antibiotic use in the community during the COVID-19 pandemic influences levels of resistance. Furthermore, it is interesting to speculate about lessons we could learn from this pandemic with respect to reducing spread of infectious diseases, influencing patient’s health care seeking behavior and appropriate levels of antibiotic use, which could all contribute to stemming the tide of antimicrobial resistance.

The main strengths of our study are the sample size, data completeness and the opportunity to compare data from the same practices during two time periods. The JGPN database encompasses well-documented electronic primary health care data of more than 70 primary care practices. In the Netherlands, characteristics of all contacts in primary care are systematically registered using ICPC codes. Still, some limitations deserve further attention. First, a direct link between an individual contact and antibiotic prescribing is missing. As such, we collected antibiotic prescriptions within two days before the start and two days after the stop dates of the episode. Although unlikely, the antibiotic could therefore have been prescribed for other reasons. Second, we have not extracted data on specialist and hospital referrals which means that our findings related to complications should be interpreted with caution. Finally, we are not able to draw any meaningful conclusions about the number of confirmed COVID-19 cases since patients were not routinely tested for SARS-CoV-2 during the first wave in the Netherlands.

It is interesting to speculate about the implications of our findings for future primary care practice. A priori, we expected a decrease in episodes, with an increase of antibiotic prescription rates during the COVID-19 pandemic. In fact, antibiotic prescription rates decreased substantially for respiratory/ear infections. This may be due to patients presenting for phone consultations with mild respiratory symptoms and GPs adhering to the national guidelines which do not recommend antibiotic treatment in uncomplicated respiratory tract infections [14]. It is not possible to confidently conclude from our data whether this lower prescription rate is safe, and whether this low rate could serve as a reasonable target in the future. It is reassuring that we did not find an increase in complications. However, we cannot rule out that some patients were not coded as complication by their GP because they were treated in out-of-hours care. Furthermore, antibiotics can shorten the duration of symptoms and are therefore recommended in current primary care infection guidelines for certain subgroups of patients [14,15]. Since duration of illness could not be captured in our routine primary care data, we are not able to draw any meaningful conclusions about patients’ symptom course.

Further research is needed to assess the longer-term impact of the COVID-19 pandemic since changes in the delivery of care over time, such as increased SARS-CoV-2 testing capacity and vaccinations might have important implications for the primary care management of common respiratory tract infections. Additionally, symptom duration and complication rates for common infections need to be captured prospectively, to assess whether decreased presentation and antibiotic use is safe.

## 4. Materials and Methods

### 4.1. Objectives

To investigate the impact of the COVID-19 pandemic on the number of infectious disease episodes and antibiotic prescribing for common infections in primary care in the Netherlands.

### 4.2. Design and Study Population

For this retrospective, observational cohort study, pseudonymized routine health care data from the JGPN were used [16]. This network covers over 70 primary care practices located in the city of Utrecht and its surrounding areas, providing care during office hours. The approximately 200 GPs from this network are experienced in using ICPC codes as part of electronic patient record keeping [17]. Participating practices and their patient population are representative of the Dutch population as a whole [16].

We included all patients enlisted in the participating practices from March through May 2019 (pre-COVID-19) and from the same period in 2020 (COVID-19 pandemic). Research with data from the JGPN is observational, without any identifiable information, and, therefore, the Medical Ethics Review Committee in the University Medical Center Utrecht does not regard such research subject to the Dutch law “Medical Research Involving Human Subjects Act”. Researchers need to conform to privacy legislation [16].

### 4.3. Data Extraction

The sizes and distributions over sex and age of the registered patient population were determined for the two time periods. From the database, contacts for common respiratory/ear, urinary tract, gastrointestinal and skin infections were extracted using the ICPC codes listed in Table A1. Infectious disease episodes were constructed by combining ICPC codes from the same chapter (R/H, U, D and S) registered within 28 days after the index contact. A new infectious disease episode thereby starts after a period of at least 28 days without any infection-related consultation of the same ICPC chapter. Age of the patient, the number and type of consultations were determined for each infectious disease episode, and antibiotic prescriptions were added. Since individual contacts and prescriptions are not directly linked in the JGPN database, antibiotic prescriptions within two days before and after the start and stop dates of the episode were captured.

### 4.4. Analyses

The following outcomes were calculated: (1) the total number of infectious disease episodes recorded from March through May for 2019 and 2020; (2) the number of episodes treated with at least one antibiotic; and (3) the antibiotic prescription rate (proportion of episodes treated with at least one antibiotic). Data were analyzed separately for respiratory/ear, urinary tract, gastrointestinal and skin infections.

MedCalc software (version 19.1.7; MedCalc Software bvba, Ostend, Belgium; https://www.medcalc.org; 2020) was used to carry out chi-square tests and to calculate the 95%-confidence intervals of relative risks. Relative risks were calculated by dividing the risk in 2020 by the risk in 2019. Risks were calculated by dividing the number of episodes that occurred during the time period of interest by the number of patients enlisted minus the number of episodes.

The course of outcomes (1) and (2) over time were determined per week for each infection type. Respiratory/ear infections outcomes (1) and (2) were determined separately per age category of 0–12, 13–40, 41–65, and older than 65 years of age. Furthermore, the number of respiratory/ear episodes with only remote (telephone) consultations were analyzed separately.

For the analysis of specific diagnoses, episodes were searched for contacts with (i) specific ICPC codes of interest: influenza-like-illness (R80, a syndromic code which does not reflect PCR-confirmed Influenza), bronchitis/bronchiolitis (R78), cough (R05), acute otitis media (H71), cystitis (U71); (ii) codes that could have been used for patients with suspected COVID-19 (R74, acute upper respiratory tract infection) or confirmed COVID-19 (in 2020 coded as R83, other respiratory tract infection); and (iii) codes for complications: pneumonia (R81), mastoiditis (H74.02), and pyelonephritis (U70).

## 5. Conclusions

In conclusion, our findings indicate that the COVID-19 pandemic has had profound effects on the number of infectious disease episodes and antibiotic use in primary care in the Netherlands. Consequently, the number of infectious disease episodes treated with antibiotics decreased. We found no evidence of an increase in complications.

## Figures and Tables

**Table 1:** Number of disease episodes and episodes with antibiotic prescription for respiratory/ear, urinary tract, gastrointestinal and skin infections in 2019 and 2020 (March through May).

	Episodes Total			Episodes with Antibiotics			Prescription Rate		
	*n*	*n*	RR (CI)	*n*	*n*	RR (CI)	%	%	% 2020 minus % 2019 (CI)
Year	2019 *	2020 *		2019	2020		2019	2020	
Resp/ear	16,672	15,580	0.90 (0.88 to 0.92) ^†^	3567	2022	0.54 (0.52 to 0.58) ^†^	21%	13%	−8.0% (−8.8% to −7.2%) ^†^
Urinary	5376	4421	0.79 (0.76 to 0.82) ^†^	2893	2540	0.84 (0.80 to 0.89) ^†^	54%	57%	3.0% (1.0% to 5.0%) ^†^
GI	2367	1338	0.54 (0.51 to 0.58) ^†^	73	58	0.76 (0.54 to 1.08)	3%	4%	1.0% (−0.2% to 2.3%) ^†^
Skin	2848	2103	0.71 (0.67 to 0.75) ^†^	892	707	0.76 (0.69 to 0.84) ^†^	31%	34%	3.0% (0.4% to 5.6%) ^†^
Total	27,263	23,442	0.83 (0.81 to 0.84) ^†^	7425	5327	0.69 (0.67 to 0.71) ^†^	27%	23%	−4.0% (−4.8% to −3.2%) ^†^

* Total numbers of patients registered in the Julius General Practitioners’ Network: 389,708 in 2019 and 405,688 in 2020. ^†^ Values with a significance level *p* < 0.05. Abbreviations: RR: relative risk; CI: confidence interval; Resp/ear: respiratory tract infections including ear infections; Urinary: urinary tract infections; GI: gastrointestinal infections; Skin: skin infections.

**Table 2:** Number of disease episodes and episodes with antibiotic prescription for respiratory tract and ear infection episodes per age category in 2019 and 2020 (March through May).

Age	Registered Patients		Episodes Total			Episodes with Antibiotics			Prescription Rate		
	*n*	*n*	*n*	*n*	RR (CI)	*n*	*n*	RR (CI)	%	%	% 2020 minus % 2019 (CI)
	2019	2020	2019	2020		2019	2020		2019	2020	
0–12	61,414	62,505	4262	2651	0.61 (0.58 to 0.64) ^†^	943	428	0.44 (0.40 to 0.50) ^†^	22%	16%	−6.0% (−7.8% to −4.1%) ^†^
13–40	156,535	166,306	4998	5166	0.94 (0.91 to 0.98) ^†^	960	530	0.52 (0.47 to 0.58) ^†^	19%	10%	−9.0% (−10.4% to −7.6%) ^†^
41–65	120,787	123,710	4555	5322	1.14 (1.10 to 1.19) ^†^	932	614	0.64 (0.58 to 0.71) ^†^	20%	12%	−8.0% (−9.5% to −6.6%) ^†^
>65	50,968	53,163	2857	2441	0.82 (0.78 to 0.86) ^†^	732	450	0.59 (0.52 to 0.66) ^†^	26%	18%	−8.0% (−10.2 to −5.8%) ^†^
Total	389,704 *	405,684 *	16,672	15,580	0.90 (0.88 to 0.92) ^†^	3567	2022	0.54 (0.52 to 0.58) ^†^	21%	13%	−8.0% (−8.8% to −7.2%) ^†^

* Four patients were left out due to unknown age. ^†^ Values with a significance level *p* < 0.05. Abbreviations: abx: antibiotics; CI: confidence interval.

**Table 3:** Number of disease episodes and episodes with antibiotic prescription for respiratory tract/ear infections and cystitis episodes in 2019 and 2020 (March through May).

Year of Episode			Cough R05	Influenza R80	Bronchitis/ Bronchiolitis R78	Acute Otitis Media H71	Cystitis U71	Acute Upper RTI R74	Other RTI Including COVID-19 R83
2019 *	Episodes Total	*n*	4194	282	582	1445	3619	3734	114
	Episodes with abx	*n*	548	30	272	675	2523	521	29
	Prescription Rate	%	13%	11%	47%	47%	70%	14%	25%
2020 *	Episodes Total	*n*	4487	466	319	562	3067	5001	831
		RR (CI)	1.03 (0.99 to 1.07)	1.59 (1.37 to 1.84) ^†^	0.53 (0.46 to 0.60) ^†^	0.37 (0.34 to 0.41) ^†^	0.81 (0.78 to 0.85) ^†^	1.29 (1.23 to 1.34) ^†^	7.00 (5.76 to 8.52) ^†^
	Episodes with abx	*n*	360	29	102	257	2223	493	118
		RR (CI)	0.63 (0.55 to 0.72) ^†^	0.93 (0.56 to 1.55)	0.36 (0.29 to 0.45) ^†^	0.37 (0.32 to 0.42) ^†^	0.85 (0.80 to 0.90) ^†^	0.91 (0.80 to 1.03)	3.91 (2.60 to 5.87) ^†^
	Prescription Rate	%	8%	6%	32%	46%	72%	10%	14%
		% 2020 minus % 2019 (CI)	−5.0% (−6.3 to −3.7%) ^†^	−5.0% (−9.6% to −1.0%) ^†^	−15.0% (−21.3% to −8.3%) ^†^	−1.0% (−5.8 to 3.9%)	2% (−0.2% to 4.2%)	−4.0% (−5.4% to −2.6%) ^†^	−11.0% (−19.9% to −3.5%) ^†^

* Total numbers of patients registered in the Julius General Practitioners’ Network: 389,708 in 2019 and 405,688 in 2020. ^†^ Values with a significance level *p* < 0.05. Abbreviations: RTI: respiratory tract infection; abx: antibiotics; CI: confidence interval.

**Table 4:** Complications, total number of episodes and episodes with antibiotic prescription.

Year of Episode			Pneumonia R81	Mastoiditis H74_02	Pyelonephritis U70
2019 **	Episodes Total	*n*	947	11	98
	Episodes with abx	*n*	592 *	5 *	62 *
	Prescription rate	%	63%	46%	63%
2020 **	Episodes Total	*n*	590	14	95
		RR (CI)	0.60 (0.54 to 0.66) ^†^	1.22 (0.56 to 2.69)	0.93 (0.70 to 1.23)
	Episodes with abx	*n*	292 *	2 *	60 *
		RR (CI)	0.47 (0.41 to 0.54) ^†^	0.38 (0.07 to 1.98)	0.93 (0.65 to 1.33)
	Prescription rate	%	50%	67%	63%
		% 2020 minus % 2019 (CI)	−13% (7.9% to 18%) ^†^	21% (−15.9% to 51.6%)	0% (−13.4% to 13.4%)

* It is likely that some patients were referred to hospital and received an antibiotic prescription there, which may not have been recorded in the primary care file. ** Total numbers of patients registered in the Julius General Practitioners’ Network: 389,708 in 2019 and 405,688 in 2020. ^†^ Values with a significance level *p* < 0.05. Abbreviations: abx: antibiotics; CI: confidence interval.

## Data Availability

Data are not publicly available due to ethical and legal restrictions.

## Supplementary Materials

The following are available online at https://www.mdpi.com/2079-6382/10/2/196/s1, Figure S1: Respiratory/ear infection episodes over time per age group (0–12 years), Figure S2: Respiratory/ear infection episodes over time per age group (13–40 years), Figure S3: Respiratory/ear infection episodes over time per age group (41–65 years), Figure S4: Respiratory/ear infection episodes over time per age group (>65 years).

## Appendix A

**Table A1:** International Classification of Primary Care (ICPC) codes used to group consultations into episodes (ear/respiratory, urinary tract, gastrointestinal and skin infection episodes).

ICPC Chapter	Months: March through May 2019 and March through May 2020
H Ear/Respiratory	H71 acute otitis media H99 ear/mastoid disease H01 earache H04 ear discharge H70 otitis externa H72 serous otitis media H74 chronic otitis media/other otitis H74.01 chronic otitis media H74.02 mastoiditis R21 throat symptoms R22 tonsil symptoms R72 strep throat R74 acute upper RTI R76 tonsillitis/tonsillar abscess R76.01 tonsillitis R76.02 tonsillar abscess R77 laryngitisR80 influenza R83 other RTI R05 cough R09 sinus symptoms R75 sinusitis R78 bronchitis R81 pneumonia R82 pleurisy R71 whooping cough R70 tuberculosis
U Urinary	U01 dysuria U02 urgency U71 cystitis U70 pyelonephritis Y73 prostatitis
D Digestive	D11 diarrhoea D70 gastrointestinal infection D73 gastroenteritis D22 parasites D92 diverticular disease
S Skin	S09 infected finger/toe S10 boil/carbuncle S11 skin infection post-traumatic S76 other skin infection S84 impetigo
