# Supplementary material for: Impact of the COVID-19 Pandemic on Antibiotic Prescribing for Common Infections in The Netherlands: A Primary Care-Based Observational Cohort Study
Source: Antibiotics (Basel). 2021 Feb 18;10(2):196. doi: 10.3390/antibiotics10020196 (PMC7922191; doi:10.3390/antibiotics10020196)
Supplement: Supplementary file 1 [file antibiotics-10-00196-s001.zip › Supplementary Materials Figures S1-S4_08-02-2021.docx]

**S**upplementary Materials: Figures S1-S4: Respiratory/ear infection episodes over time per age group

**Figure 1.** Respiratory/ear infection episodes over time per age group (0–12 years).

**Figure 2.** Respiratory/ear infection episodes over time per age group (13–40 years).

**Figure 3.** Respiratory/ear infection episodes over time per age group (41–65 years).

**Figure 4.** Respiratory/ear infection episodes over time per age group (>65 years).
