# Supplementary figures and images for: Impact of the COVID-19 Pandemic on Antibiotic Prescribing for Common Infections in The Netherlands: A Primary Care-Based Observational Cohort Study
Source: Antibiotics (Basel). 2021 Feb 18;10(2):196. doi: 10.3390/antibiotics10020196 (PMC7922191; doi:10.3390/antibiotics10020196)

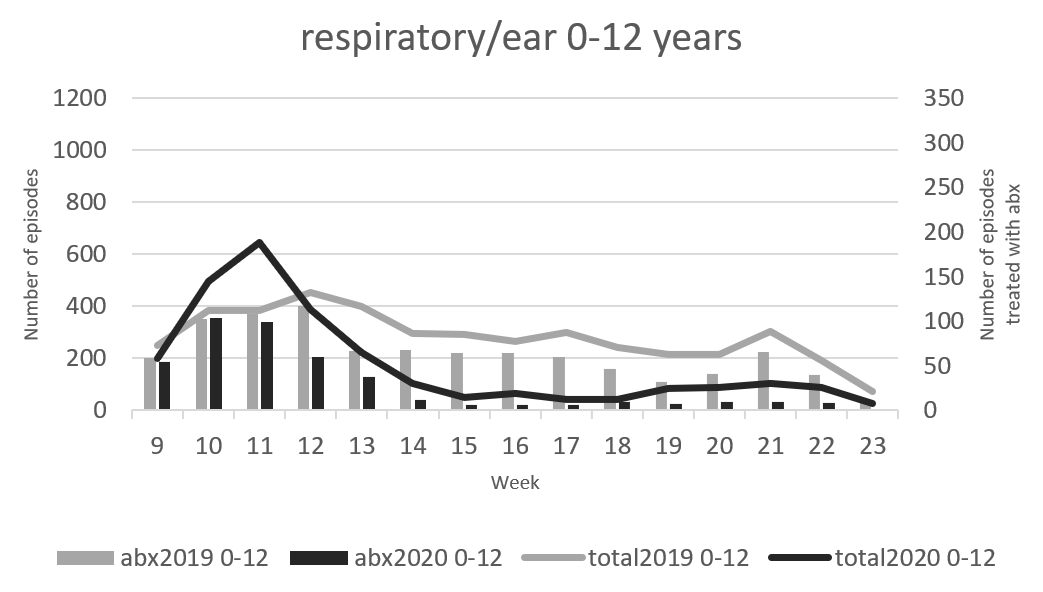

Supplement: Supplementary file 1 [file antibiotics-10-00196-s001.zip › Figure S1.png]

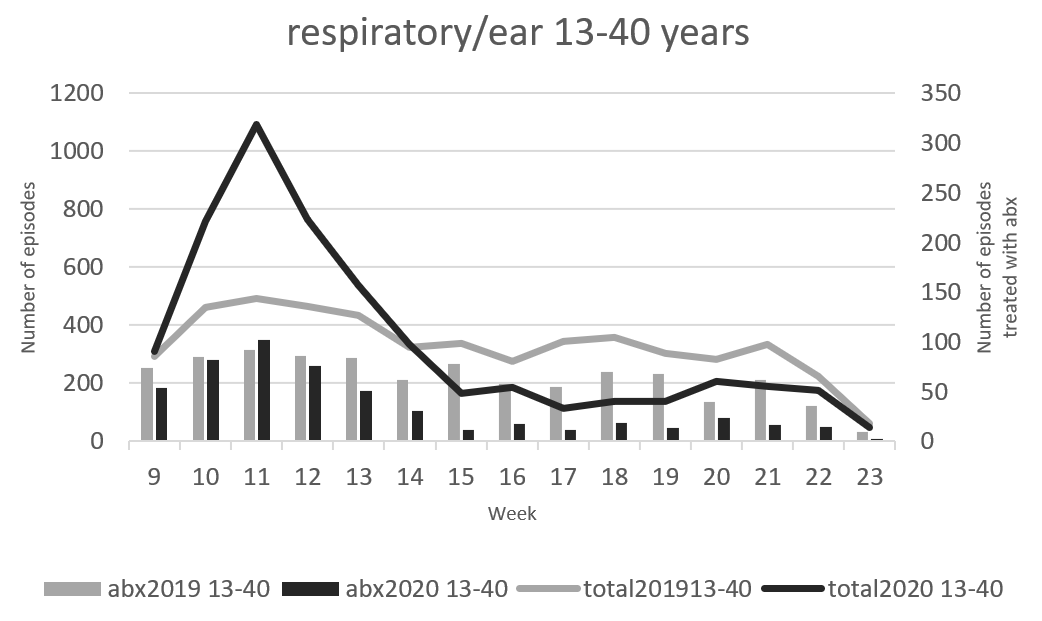

Supplement: Supplementary file 1 [file antibiotics-10-00196-s001.zip › Figure S2.png]

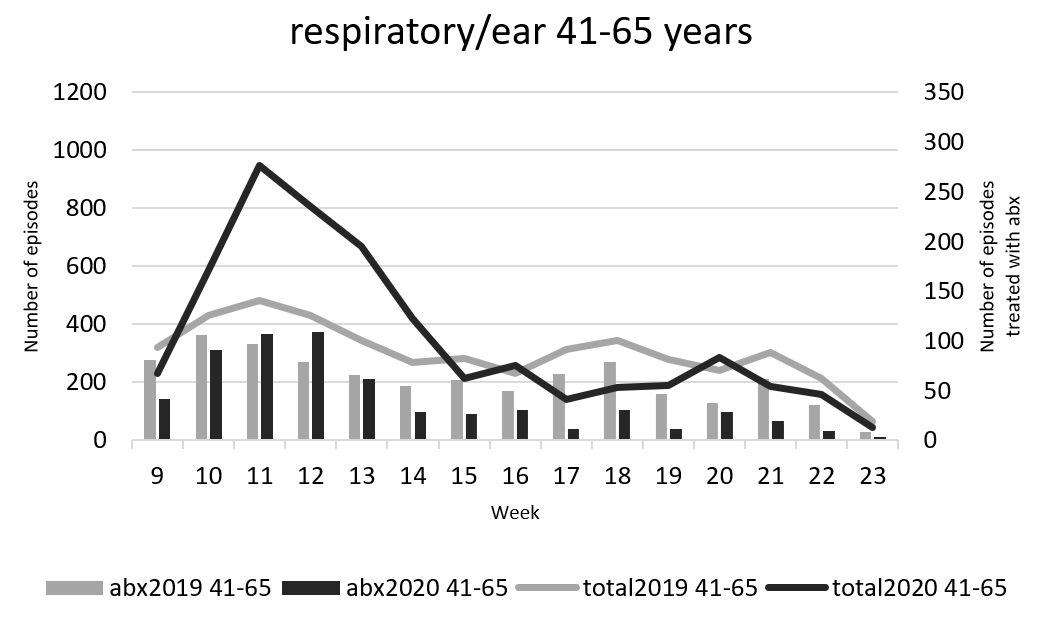

Supplement: Supplementary file 1 [file antibiotics-10-00196-s001.zip › Figure S3.png]

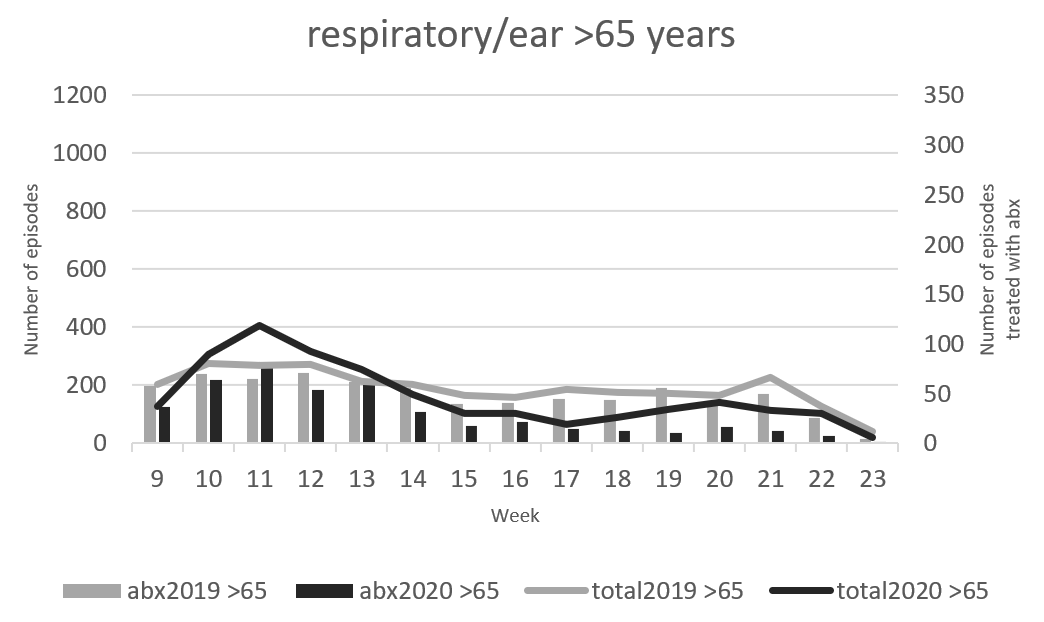

Supplement: Supplementary file 1 [file antibiotics-10-00196-s001.zip › Figure S4.png]
